# The challenge for genetic epidemiologists: how to analyze large numbers of SNPs in relation to complex diseases

**DOI:** 10.1186/1471-2156-7-23

**Published:** 2006-04-21

**Authors:** A Geert Heidema, Jolanda MA Boer, Nico Nagelkerke, Edwin CM Mariman, Daphne L van der A, Edith JM Feskens

**Affiliations:** 1 Centre for Nutrition and Health, National Institute for Public Health and the Environment, PO Box 1 3720 BA Bilthoven, The Netherlands; 2 Department of Community Medicine, United Arab Emirates University, PO Box 17172 Al Ain, UAE; 3 Functional Genomics, Maastricht University, PO Box 616 6200 MD Maastricht, The Netherlands; 4 Division of Human Nutrition, Wageningen University and Research Centre, PO Box 8129 6700 EV Wageningen, The Netherlands

## Abstract

Genetic epidemiologists have taken the challenge to identify genetic polymorphisms involved in the development of diseases. Many have collected data on large numbers of genetic markers but are not familiar with available methods to assess their association with complex diseases. Statistical methods have been developed for analyzing the relation between large numbers of genetic and environmental predictors to disease or disease-related variables in genetic association studies.

In this commentary we discuss logistic regression analysis, neural networks, including the parameter decreasing method (PDM) and genetic programming optimized neural networks (GPNN) and several non-parametric methods, which include the set association approach, combinatorial partitioning method (CPM), restricted partitioning method (RPM), multifactor dimensionality reduction (MDR) method and the random forests approach. The relative strengths and weaknesses of these methods are highlighted.

Logistic regression and neural networks can handle only a limited number of predictor variables, depending on the number of observations in the dataset. Therefore, they are less useful than the non-parametric methods to approach association studies with large numbers of predictor variables. GPNN on the other hand may be a useful approach to select and model important predictors, but its performance to select the important effects in the presence of large numbers of predictors needs to be examined. Both the set association approach and random forests approach are able to handle a large number of predictors and are useful in reducing these predictors to a subset of predictors with an important contribution to disease. The combinatorial methods give more insight in combination patterns for sets of genetic and/or environmental predictor variables that may be related to the outcome variable. As the non-parametric methods have different strengths and weaknesses we conclude that to approach genetic association studies using the case-control design, the application of a combination of several methods, including the set association approach, MDR and the random forests approach, will likely be a useful strategy to find the important genes and interaction patterns involved in complex diseases.

## Background

The field of genetic epidemiology aims to identify genetic polymorphisms involved in the development of diseases. Single-locus methods measure the effect of one locus irrespective of other loci and are useful to study genetic diseases caused by a single gene, or even loci within single genes. To study complex diseases such as cardiovascular disorders or diabetes single-locus methods may not be appropriate, as it is possible that loci contribute to a certain complex disease only by their interaction with other genes (epistasis), while main effects of the individual loci may be small or absent [1]. Single-locus methods can not detect complex patterns [2], thus underestimate the genetic contribution to disease in the presence of interactions between loci. Therefore, approaches have been developed that take into account that complex diseases can be caused by an intricate pattern of genetic variants. These approaches are referred to as multi-locus methods and are specifically designed to find multiple disease loci, possibly on different chromosomes [3]. Diseases with a polygenic background can be studied by multi-locus methods, but also multi-factorial diseases by incorporating environmental predictors into the model.

Studying the effect of multiple genetic and/or environmental predictors and their interactions is fraught with statistical problems. One of these problems involves multiple testing. For each tested locus the probability to make a type I error is present, which is the probability to accept the hypothesis that the locus has an effect while in reality it does not. By testing multiple markers independently the type I error probability of finding a false positive result is increased. Two correction procedures for multiple testing are Bonferroni procedure and the false discovery rate [4]. Adjusting for multiple testing leads to a decrease of power (the probability to detect an effect when the effect is present) which makes it less likely to find weak genetic effects. Several multi-locus methods, discussed later in this commentary, have been developed to solve the multiple testing problem. These methods have greater power to detect susceptibility loci than single-marker tests.

The problem of modest sample sizes to test interactions for a large group of predictors (high-dimensional data) is referred to as the 'curse of dimensionality' problem [5]. The number of observations becomes too small relative to the number of predictors tested as few or no observations for combinations of predictors will occur. Traditional parametric approaches suffer from the dimensionality problem as it results in inaccurate parameter estimates for interaction effects [6]. Multi-locus methods are needed to select from the large amount of genetic and environmental predictors a small group of predictors and/or interactions between predictors that have a significant effect on the disease outcome. Subsequently, parameters for the selected predictors can be estimated by logistic regression analysis.

A third problem in the analysis of the effect of multiple genetic and environmental predictors on disease is the presence of correlated predictors in the dataset. An example is the presence of SNPs that are in linkage disequilibrium (LD) among the set of SNPs tested for association with disease. The power of a method to detect important predictors can be decreased when correlated predictors are tested. Some of the multi-locus methods discussed in this commentary are able to handle correlated predictors. Very high correlations between predictors, which is referred to as multicollinearity, is always a problem for methods: highly correlated predictors have an equal chance to be selected and one predictor may falsely be selected instead of the highly correlated predictor that is truly associated with disease. Multicollinearity can be coped with statistically by combining data from multiple predictors into a single variable [7], for example combining SNPs that are in high LD into haplotypes.

Another difficult problem is the presence of heterogeneity [8]. Genetic heterogeneity is present if different genetic loci are independently associated with the same disease. The genes in which these loci are present can be part of different etiological pathways leading to the same disease or be part of the same pathway. Irrespective of the biological mechanism that gives rise to genetic heterogeneity, the association of these loci with the disease will be reduced if the total sample is used for measuring the association. A method that is not robust in the presence of genetic heterogeneity will likely suffer from a decrease in power to detect genetic effects. If genetic heterogeneity is not handled it can be accounted for by employing cluster analysis of genetic markers to identify groups of individuals with similar genetic profiles [8]. If clusters are present, association analyses of markers with the outcome variable should be accommodated for cluster effects [9]. Another form of heterogeneity that can affect the power to detect markers associated with disease is the presence of phenocopies. Phenocopies are individuals affected by the disease while they have a low-risk genotype profile. These individuals have developed the disease due to certain environmental factors. As in the presence of genetic heterogeneity, phenocopies will decrease the association between genetic markers and the disease if the association is studied using the total sample. Cluster analysis of environmental factors in the population can be used to define subgroups and cluster effects should be taken into account in the association analyses.

**Figure 1:**
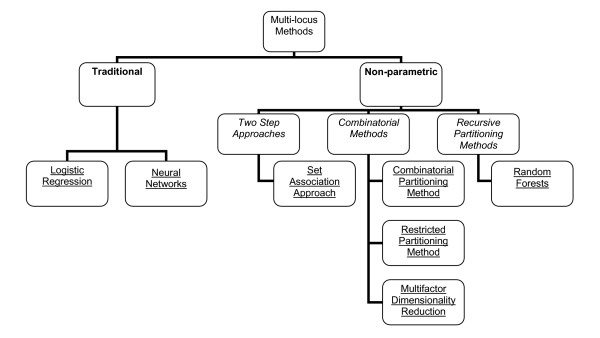
Diagram containing the different methods described in this commentary.

Many genetic epidemiologists have collected data on large numbers of genetic markers but are not familiar with the available methods to assess their association with complex diseases. In this article we review the strengths and weaknesses of methods for analyzing the genetic and/or environmental effects on disease or disease-related variables. These methods are presented in figure 1. Logistic regression and neural networks are discussed so as to compare non-parametric methods with these more 'traditional' statistical methods. The non-parametric methods have been selected as several genetic association studies have been conducted using these methods to analyze their data. This field is rapidly in progress and more methods are becoming available. This commentary does not pretend to cover all available multi-locus methods, nor to provide their statistical background, but aims to function as a starting and reference point for researchers in the field of genetic epidemiology who want to become more acquainted with multi-locus methods.

## Method of evaluation

To provide an overview of the strengths and weaknesses of each method the ability of the methods to model the effects of multiple genetic and/or environmental predictors on disease outcome is evaluated for the following features:

- are the methods able to handle large numbers of predictors relative to the number of observations (dimensionality problem);

- number of predictors that can be analyzed in modest sample sizes;

- power to detect genetic effects;

- how do the methods handle interactions;

- do the methods maintain power if correlated predictors are present in the dataset;

- performance of the method if genetic heterogeneity is present;

- software availability and whether available software is open-source.

For each method a description is given followed by the discussion of the performance of the method for the different features. First, logistic regression is discussed, followed by neural networks, the set association approach, the combinatorial methods and the random forests approach. After the discussion of the different methods, strengths and weaknesses of these methods are compared and a strategy to analyze the effect of multiple genetic and environmental predictors on disease is proposed. As many of these methods use permutation testing to determine the statistical significance of predictors a short explanation of this test will be given here.

For testing the statistical significance of the association between selected predictors and the outcome variable permutation tests are used to obtain the distribution of the test statistic under the null hypothesis of no association. Permutation tests generate many samples for which the association between the predictors and the outcome variable has been disrupted by randomly distributing the values of the predictors or outcome variable over the observations. For each permuted sample the method is applied to calculate the test statistic and together these test statistics form the distribution of the test statistic under the null hypothesis. The proportion of permutation samples with a value exceeding the value of the test statistic of the observed data gives the significance level for the observed test statistic [10].

To evaluate the ability of a model to classify and predict a certain outcome variable, multi-fold cross-validation is often used. This procedure will be explained because different methods use multi-fold cross-validation to obtain the classification and prediction error of models relating predictors to a certain outcome variable. In multi-fold cross-validation the data are randomly divided into groups of approximately the same size. The parameters of the model are estimated by all groups except for one, this remaining group is used for obtaining the prediction error (or prediction accuracy) of the model. As an example, a ten-fold cross-validation divides the data into ten groups of equal size. Nine groups are used to build the model. For quantitative traits, a fraction of the prediction error of this model is computed by the remaining group. By turns the ten groups are used to compute a fraction of the prediction error and the sum of the ten fractions forms the prediction error. For categorical outcome variables (e.g. disease status) the prediction error is calculated for each of the ten groups.

To reduce arbitrariness in the division of the data into the different groups when estimating the expected (average) prediction error, the multi-fold cross-validation is repeated several times. Each time the data is randomly divided into the same number of groups. For quantitative traits the sum of the prediction errors obtained by the different cross-validations divided by the number of repeats gives an average prediction error. For dichotomic traits the average prediction error is the sum of prediction errors over all groups divided by the number of groups. The average prediction error is an unbiased estimate of the prediction error of the model.

## Traditional methods

### Logistic regression

A parametric statistical method often applied in genetic epidemiology is logistic regression. It is used to analyze the effect of genetic and environmental predictors on a dichotomic outcome, for example disease status. Predictors are linked to the outcome variable by the logit function. While many methods can be used to test for an association between predictors and disease in case-control studies, in such case-control studies logistic regression is the only appropriate method to consistently estimate the strength of association between a predictor and disease [11]. The conditional logistic regression (CLR) method is appropriate if stratification is present in the data, for example in a study design with matched cases and controls. CLR adjusts for the matching of the cases and controls by stratifying the matched case-control pairs.

### Features of the logistic regression method

One of the disadvantages of the logistic regression method is that it performs poorly in the presence of the dimensionality problem; it may lead to false positive results [12] and a low power to detect interactions [6]. This may be overcome by stepwise regression analysis, which reduces the large number of predictors to a smaller number of predictors that are significantly related to disease. With forward selection, significant main effects and interactions between these main effects are included in the model. With backward selection, non-significant effects are excluded from the full model containing all parameters. There are drawbacks to the use of these standard selection procedures. With forward selection, interactions can only be tested for the main effects included in the model. Backward selection has the disadvantage that it cannot properly work in the presence of too many variables relative to the number of cases. Even if it does work, inclusion of too many parameters reduces the power of the model. Applying the least absolute shrinkage and selection operator (LASSO) [13] for selection of predictors in logistic regression may be more useful than standard selection procedures. This procedure shrinks the coefficients of predictors that are not important to zero, thereby selecting a subset from a larger number of predictors. It appears to have a better performance than standard backwards selection, but one disadvantage of the LASSO may be that it does not reduce the number of predictors substantially [14]. Therefore, for selection of important predictors it will also be useful to apply other selection methods before using logistic regression analysis to estimate the strength of association between selected predictors and disease.

Correlation between predictors may be a problem for logistic regression as different model building strategies may lead to different results [7]. Also, logistic regression does not handle genetic heterogeneity well as it models the relation between predictors and risk of disease for all individuals in the sample [15] and therefore it does not account for the presence of subgroups with different relationships between disease and genetic make-up. If different subsets of genes work in different subsets of the sample then logistic regression will probably not detect the different genetic causes of disease [16]. To perform logistic regression analysis many standard software packages (e.g. SAS, SPSS) are available.

### Neural networks

Artificial neural networks are used to recognize patterns in the observed data and can be applied to determine genetic and environmental predictors related to disease. In genetic epidemiology, neural networks can be used to select SNPs that may contribute to disease.

In this section we will describe in the first part the structure of a network that is commonly used (the feed forward network) and how neural networks usually are applied to obtain the best structure. In the second part we describe the parameter decreasing method [17], which can be used to select a subset of important predictors among a larger set of predictors. The genetic programming optimized neural network (GPNN) [18] is a strategy that will be described separately in the third part as it optimizes the structure of the network in a different way and different steps are involved to select the best model.

### Structure of the feed forward network

A type of network commonly used consists of an input layer, one or more hidden layers and an output layer. Each layer is built up of nodes whereby one layer of nodes is connected to the next layer and weights are assigned to the connections. For example, with 10 input nodes, 6 hidden layer nodes and 1 output node the number of connections, and thus weights, equals 10*6 + 6*1 = 66. This type of network has a feed-forward structure: the flow of information is from the input layer, via the nodes of the hidden layer(s) to the node(s) of the output layer. The values of the predictors are the input values for the neural network. The combined input values are processed by each of the nodes of the hidden layer by a transfer function. For dichotomic outcome variables the transfer function is for example the logistic function. A network containing one hidden layer node with a logistic transfer function is equivalent to logistic regression analysis [19] and networks containing more hidden nodes with logistic transfer functions are generalizations of logistic regression to more complex nonlinear relationships between predictors and disease [20]. These non-linear relationships do not need to be defined. More layers and nodes increase the complexity of the model which enables the network to model complex interactions between the predictor variables. Networks fall in between parametric and non-parametric approaches as they provide large but not unlimited numbers of parameters to analysis methods [20].

The output of each node is determined by the outcome of the transfer function and is processed by each node of the next hidden layer (if present). The output of the last hidden layer is processed by the output node. The network associates the input values of the predictors with the output values given by the network. The amount of error between the output values of the model and the observed values is measured by an error function, for example a sum squared error.

Training the network, i.e. essentially estimating all the (hidden) parameters in the transfer function, is the process of adjusting the weights of the connections whereby weights are increased if they improve the output values and decreased if they result in more error. The procedure to optimize the weights is referred to as the back propagation algorithm [19]. The aim of the training is to obtain the model containing weight values that minimize the classification error of the network. Multi-fold cross-validation is used to divide the data into a training set and an evaluation set. The network model is constructed using the training set, the evaluation set is used to obtain the prediction error of the model. The error between the predicted values and observed values of the evaluation set gives the prediction error of the network. Each group created by multi-fold cross-validation is used to obtain the prediction error and the average prediction error is given by the sum of the prediction errors divided by the number of groups. The best model is the model with the lowest classification and prediction error. After the model has been obtained, predictors associated with the disease can be selected.

### Parameter decreasing method

To select important SNPs from the total group of SNPs that were used to construct the network model, a parameter decreasing method (PDM) can be used [17]. The procedure of PDM starts by deleting one SNP from the total number of SNPs and constructs a model containing the remaining SNPs. In turn each SNP is deleted from the total number of SNPs and with the remaining SNPs a model is constructed. From the constructed models the model with the lowest number of misclassified subjects in both the training and evaluation set is selected. This process is repeated until one SNP remains. For each selected model a measure of prediction accuracy is calculated by the sum of true predicted cases and controls divided by the total number of the evaluation sample. The prediction accuracy is calculated for each evaluation set created by multi-fold cross-validation and the sum of the prediction accuracies divided by the number of evaluation sets gives the average prediction accuracy.

The PDM has been applied to select from 25 SNPs a subset of susceptible SNPs of childhood allergic asthma [17]. The average prediction accuracy started to decrease after SNPs were excluded from the model containing 10 SNPs. To minimize the effect of randomized initial weight values, five PDM trials were performed and the importance of SNPs that remained in the last 10 SNPs of each trial was determined. For each trial the 10 SNPs were ordered from 1 to 10, based on the significance level of each SNP with the disease. The sum over the five trials for the SNPs that remained in the different trials was computed (sums can range from 1 to 50) and it is assumed that SNPs with higher scores are more important. The selected SNPs were used to construct models in order of importance of SNPs and for each model the prediction accuracy was calculated. Models with 10 of the most important SNPs or more had high prediction accuracy. The model containing the 10 most important SNPs had the same prediction accuracy as the model containing all 25 SNPs.

A permutation test can be applied to determine whether at least one of the selected SNPs is associated with the disease by randomly permuting the values of the selected SNPs [21].

To investigate important interactions, Tomita et al. [17] computed for 2-SNP and 3-SNP combinations the p-values by χ^2^-test and selected SNP combinations with a p-value lower than 0.05. Combinations obtained in this manner likely contain false positive results because correction for multiple testing has not been applied. Therefore they used another measure of evaluation which they refer to as the effective combination value (ECV). If SNPs in a combination are independent, then the product of their separate p-values is equal to the p-value of the combination. ECV is the ratio of a SNP combination p-value divided by the product of SNP p-values and ECV < 1 suggests that interaction is present. SNP combinations that meet criteria for both χ^2 ^p-values and ECV values are selected.

### Genetic programming optimized neural networks

A different strategy which can be used to select predictors associated with disease is referred to as genetic programming optimized neural networks [18]. Ritchie et al. [18] developed this strategy to optimize the neural network structure in order to improve selection of disease associated predictors. The back propagation algorithm described in the first part of the neural network section optimizes the weights. GPNN on the other hand not only optimizes the weights, but also a set of inputs that is selected from a larger set of predictors, the number of hidden layers and the number of nodes within the hidden layer(s). Cross-validation is also applied in GPNN to obtain for each partition of the data the best model and the prediction error for this selected model.

The genetic programming procedure starts with random selected models and evolves during the process to the model with the best structure. The steps taken by GPNN to obtain the best model are described here, more detailed information can be found in [18,22]. First, a sample of all possible different GPNN models is randomly generated, using for each model a random subset of predictors from the total number of predictors. These initial GPNN models may differ in size. For each of the generated models is determined how well it fits the data, for example by its classification error. From these models a new generation of models is formed, which is equal to the number of models that were generated at the start of the process. This new generation of models is formed by directly copying a predefined proportion of the best models (those with the lowest classification error if classification error is used as fitness function) as well as by exchanging different parts between the models for another subset of best models. Thus, compared to the previous generation the new generation consists of similar models (the best proportion of models of the previous generation) and new models that are the result of recombining models of the previous generation (which is another subset of best models than the models that were copied). The size of the recombined models is allowed to change. The new generation of models replaces the previous generation and the process is repeated, bringing forth a next generation of models. This process continues until GPNN reaches a certain criterion (for example a classification error of zero or the maximum number of generations specified by the researcher). The model in the last generation that has the best fit (e.g. lowest classification error) is denoted as the best GPNN model and the prediction error for this model is determined by the remaining part of the data. For each partition created by cross-validation a best GPNN model with the corresponding prediction error is obtained. For example, 10-fold cross-validation will result in 10 best GPNN models.

To determine the importance of predictors or predictor combinations, a cross-validation consistency measure can be used, which is the number of times a predictor or predictor combination is selected in a best model across all validation sets, divided by the number of validation sets. The predictor or predictor combination which has the highest cross-validation consistency is denoted as the final selected model.

An example of GPNN application to case-control data is the study of Motsinger et al. [22] on Parkinson's disease.

### Features of neural networks

The advantage of neural networks over logistic regression is the possibility to flexibly model complex relationships between the predictor variables and the disease status. Tomita et al. [17] compared the prediction accuracy of constructed models of neural networks with logistic regression analysis for the models containing 25 and 10 SNPs. Constructed models by neural networks had high prediction accuracy while the accuracy was low for logistic regression analysis. A disadvantage of the PDM is that a cut-off value for the prediction accuracy to select SNPs as susceptible is not given.

In general, as the network can handle a limited number of predictor variables depending on the number of observations in the dataset, faced with testing very large numbers of genetic markers the network is subject to the dimensionality problem [3]. GPNN however is not subject to the dimensionality problem because it uses only a random selection of predictors to build the initial GPNN models and selects the most important predictors during the process.

Studies investigating the power for neural networks using PDM have not been found in the literature, thus information about the power of the PDM to detect important effects is not available at the present time. For GPNN, the power to detect important SNPs in the presence of unrelated SNPs is higher compared to the commonly used feed forward NN using a back propagation algorithm [18]. Using simulated data, Motsinger et al. [22] showed that the power of GPNN to detect gene-gene interactions in two and three locus interaction models is high. The number of unrelated SNPs included however was not large and further information on the power of GPNN to detect genetic effects among a large set of unrelated SNPs is needed.

If important interactions between SNPs are present, PDM will likely be able to detect the SNPs involved in the interaction, because deleting a SNP would have an effect on the prediction accuracy. Important interactions between SNPs will therefore lead to selection of these SNPs. Also, most of the 2-SNP and 3-SNP combinations identified by Tomita et al. [17] were combinations of SNPs that had been selected by the PDM procedure, followed by combinations of selected and unselected SNPs and the least number of combinations was found for unselected SNPs. This suggests that neural networks are able to select SNP combinations accurately [17].

For detection of the genetic polymorphisms involved in disease, correlated markers are a problem for neural networks using the PDM. If one marker is associated with disease, but is correlated with another marker, deleting the marker associated with disease will result in a smaller decrease in the value of the prediction accuracy compared to a situation of uncorrelated markers. Therefore, the power to detect the association of the risk marker with the disease will be reduced if this marker is correlated with one or more other markers. The power of GPNN to detect important predictors will not be reduced when correlation between predictors is present. GPNN models containing important predictors are more informative and will have lower classification errors than models containing predictors correlated with the important predictors. Important predictors will therefore be selected during the process.

Neural networks can determine substructures within a data set which enables them to handle genetic heterogeneity [20]. Software to perform neural network analysis of case-control data using PDM is freely available [23], the software is however not open-source. At the moment, software for GPNN is not available.

## Non parametric methods

### Two step approaches

Several genetic association studies have employed the two step approach, which consists of the following two steps:

- Step 1: determine a small number of potentially important markers;

- Step 2: model interactions between important markers and/or environmental predictors.

In the first step a non-parametric approach is applied to reduce many markers to a small number of important markers. For the second step environmental predictors can be introduced to the model and logistic regression or neural networks can be used to test gene-gene and/or gene-environment interactions. In the two step approach coupled-logistic regression can be applied to analyze interactions between the selected markers obtained in the first step [24,25]. The coupled-logistic regression procedure first uses one forward selection step to model the two-way and higher-order interactions between the selected markers and environmental predictors if included. Then backward selection is employed to eliminate non-significant interactions.

### Set association approach

A non-parametric approach for selecting a set of important markers as a first step is the set association approach. The set association approach is described in this section; more detailed information can be found in [26]. Instead of categorical predictors such as marker genotypes, the set association approach can also be used to analyze the effect of quantitative predictor variables [27].

The set association approach starts by calculating a test-statistic for each marker separately, which is a product of two test statistics. The first statistic measures the association of a marker with disease outcome. As measure of association χ^2 ^can be calculated from the contingency table of alleles (or genotypes) with disease status, but other statistics can be used as well. The deviation of a marker from the null-hypothesis of Hardy-Weinberg (HW) equilibrium is used as the second test-statistic, which is chi-square distributed. χ^2 ^values for deviations from HW equilibrium are calculated in the case group and larger deviations indicate an association between the marker and the disease. Very large χ^2 ^values for HW disequilibrium in the control group can indicate genotyping errors. To correct for the quality of genotyping, markers showing large χ^2 ^values in controls (e.g. χ^2 ^values exceeding the χ^2 ^value corresponding to the 99-th percentile) are deleted or set to zero [26]. Thus, for the calculation of the test-statistic for each marker, information is used from allelic association, deviation from HW equilibrium and genotyping errors.

Subsequently, the markers are ordered based on their value for the test-statistic. The set association approach starts with the selection of the marker with the largest test statistic and calculates sum statistics by adding each time the most important marker from the group of unselected markers. Increasing sums of markers are formed and the number of markers in the sums ranges from 1 to a predefined maximum number of M markers, for example 20. The significance level of each sum of markers is tested using a permutation test. The set association approach uses, and holds fixed, the observed genotypes, but randomly permutes the variable that indicates the disease status. Many permuted samples are formed and for each sample sum-statistics are calculated. The p-value for a certain sum of markers represents the proportion of permuted samples exceeding the value of the sum of markers of the observed sample. Instead of testing many markers, M sums (e.g. 20) are tested. Increased number of markers in the sum with an association with disease will lower the significance level of the sum. At a certain point the significance level will no longer decrease but increase as markers not contributing to disease are added to the sum. Therefore, from the M sums tested the set of markers with the lowest significance level is selected as the best set of markers. This p-value is defined as test statistic and is evaluated by a second permutation test testing the null-hypothesis of no association of the selected markers with the disease-outcome. The second round of permutation results in an overall p-value reducing the testing of M sums to one sum. The multiple testing problem that arises due to testing many markers has been overcome at this stage of the set association approach.

Applications of the set association approach have been reported for case-control studies on heart disease [27] and Alzheimer's disease [28]

### Features of the set association approach

The set association approach manages the dimensionality problem by reducing the number of markers to a smaller number of important markers. This method also provides an overall significance level for the selected markers. In general, the main advantage of two step approaches is that large numbers of markers can be evaluated for their importance in contributing to disease. Compared to the Bonferonni and the False Discovery Rate procedures that correct for multiple testing, the set association approach has more power to identify genes involved in disease; sum statistics are compounds of marker main effects which have a better performance than approaches that test each marker independently [29]. Furthermore, the power of the set association approach is enhanced by using information from allelic association, deviation from HW equilibrium and genotyping errors.

The main disadvantage of the set association approach is that genetic interactions are only tested for the markers that are selected in the sum. Important interactions with weak main effects will be missed.

To handle correlations between markers, Wille et al. [29] proposed a method to adjust the test-statistic of a marker for the correlation with the markers that are already present in the sum. Using unadjusted test-statistics, markers could be included in the sum while these markers are correlated with markers already contained in the sum. If the correlation between markers concerns non-susceptibility loci it will result in an overrepresentation of non-susceptibility loci in the sums, reducing the power of the approach. By using adjusted marker statistics the power of the test is re-established.

Genetic heterogeneity will affect the performance of the set association approach to identify important markers as this approach tests the association of markers with disease for the whole sample. Individuals affected due to different loci decreases the association between each of the loci with the disease and will result in a reduction of power of the set association approach to detect these loci. The set association approach is implemented in the program Sumstat [26] which is freely available and open-source [30]. At the moment, the adjustment of marker statistics for their correlation with markers already included in the sum has not been implemented in the software.

### Combinatorial methods

Combinatorial methods search over all possible factor combinations to find combinations with an effect on an outcome variable. The combinatorial methods that will be discussed are the combinatorial partitioning method (CPM), the restricted partitioning method (RPM) and the multifactor dimensionality reduction method (MDR). Respectively, CPM and RPM have been described more extensively by Nelson et al. [31] and Culverhouse et al. [32]. Several recent reviews are available for MDR [33,34]. CPM and RPM aim to identify factor combinations that explain best the variance of a quantitative phenotype. MDR classifies factor combinations as having a low risk or high risk on disease based on the presence of these combinations in cases versus controls. Both CPM and MDR use multi-fold cross-validation to select the factor combinations that have the best prediction of the outcome variable and to compute the average proportion of variability explained (CPM) or average prediction accuracy (MDR), which is used to evaluate the validity of the obtained factor combinations. It is important to evaluate the validity of the model to verify whether the combinations do not present false positive results but are truly associated with the disease [35].

### Combinatorial partitioning method

CPM can be used to study the effect of factor combinations on a quantitative phenotype. This phenotype can be a variable underlying the disease of interest. An example is to study factor combinations involved in the phenotype blood pressure, which underlies cardiovascular disease. To test whether a locus has an effect on a quantitative phenotype, analysis of variance (ANOVA) could be used. It performs an overall test of the differences between the mean phenotypic values of genotypes. However, with many genotypes a posteriori testing the significance of the differences between genotype means leads to the problem of multiple testing. CPM has the advantage that it determines the loci combinations with an effect on a quantitative phenotype and at the same time defines groups of genotypes with similar phenotypic means [31]. In the CPM a group of genotypes with similar phenotypic means is referred to as a genotypic partition. Combinations of two or more partitions make up a set of genotypic partitions. CPM selects sets of genotypic partitions (consisting of multi-locus genotypes) that predict variation of the quantitative trait [31]. The CPM consists of three steps:

- Select loci combinations from all loci studied. For these loci combinations, combine genotypes with similar phenotypes into partitions. Select from the total group of partitions each combination of genotypic partitions (thus each set) that predicts a certain level of variance;

- Validate each selected set by multi-fold cross-validation;

- Select the most predictive sets and make inferences about the combinations of loci and the genotype-phenotype relationships.

In the first step the combinatorial partitioning method selects all possible subsets of loci from the total group of loci that is studied. For example, if 10 loci are studied and all 2-loci combinations are considered, the number of subsets of loci examined is equal to (102)
 MathType@MTEF@5@5@+=feaafiart1ev1aaatCvAUfKttLearuWrP9MDH5MBPbIqV92AaeXatLxBI9gBaebbnrfifHhDYfgasaacH8akY=wiFfYdH8Gipec8Eeeu0xXdbba9frFj0=OqFfea0dXdd9vqai=hGuQ8kuc9pgc9s8qqaq=dirpe0xb9q8qiLsFr0=vr0=vr0dc8meaabaqaciaacaGaaeqabaqabeGadaaakeaadaqadaabaeqabaGaeGymaeJaeGimaadabaGaeGOmaidaaiaawIcacaGLPaaaaaa@310C@ = 45 pair wise combinations. For each subset of loci all genotypic partitions are examined. For two SNPs at autosomal loci the number of genotype combinations equals nine and the number of genotypic partitions investigated ranges from two till nine. A set can for example consist of two genotypic partitions, one partition containing the multi-locus genotypes AAbb, AaBb and aabb and the other partition containing the remaining genotypes. CPM evaluates all possible sets of genotype partitions and selects sets based on two criteria. The first criterion is the proportion of phenotypic variability explained by a set. For each selected set the variability between partitions should be much higher than within the partitions because these sets will explain the largest proportion of variance of the quantitative phenotype. The other criterion used is the number of individuals in a set. Only a few individuals will be present for genotypes with low frequency alleles and consequently for partitions in which these genotypes are present. Small numbers for genotypic partitions leads to unreliable estimates of the partition means and partition variance. When the number of individuals is set too low, spurious effects may be found by chance. On the other hand, genotypic partitions that do have an effect could be discarded from further analyses when the number of individuals is set too high [31].

In the second step each selected set is validated by the multi-fold cross-validation method. For validation of the selected sets all the groups generated by the cross-validation method, except for one, are used to estimate the means of the genotypic partitions of a set. The remaining group is used to compute the within partition sum of squares, which is the predicted error for this group only. The sum of the fractions of the different groups gives the total predicted error of a set. If the multi-fold cross-validation is repeated several times, an average predicted error can be calculated. From this averaged predicted error the proportion of variability explained by a set is computed, which is a measure of the predictive ability of the phenotype by a set of genotypic partitions. Sets with smaller proportions of within sum of squares explain more variability of the quantitative phenotype and thus have a higher predictive ability.

Based on the results of the cross-validation, the most predictive sets are selected in the third step. It is useful to select more than one predictive set of genotypic partitions, because by comparing the different sets more insight in the relation between combinations of loci present in these sets and the quantitative trait can likely be gained. To obtain the statistical significance of the most predictive set selected a permutation test can be performed. Phenotypic outcomes are randomly assigned to the genotypes and for each permutation sample the CPM is performed. The null-hypothesis tested is that the most predictive set is not significantly associated with the quantitative trait. The proportion of sets exceeding the observed value of proportion of variability explained by the most predictive set results in a p-value for the most predictive set.

CPM has been applied in studies of plasma triglyceride levels [31], plasma PAI-1 levels [36] and the relationship between plasma t-PA and PAI-1 levels [37].

### Restricted partitioning method

To overcome the computationally intensive search technique used by CPM, Culverhouse et al. [32] developed the restricted partitioning method. Where CPM searches over all possible combinations, RPM restricts its search in order to avoid evaluation of genotype partitions that will not explain much of the variation. The reasoning is that a group consisting of genotypes for which the difference between their mean values is large (thus having a large within group variance), will not explain much of the total variance of the quantitative trait and can therefore be discarded for evaluation. The search procedure that is used by RPM to select genotypic partitions consists of the following steps:

- Using a multiple comparison test, examine whether significant differences between mean values of genotype groups are present (at the start of the analysis each group consists of one multi-locus genotype);

- from all the non-significant pairs of genotype groups, combine the pair with the smallest difference between their mean values into a new group, thereby reducing the number of genotype groups to be evaluated with one;

- the procedure is reiterated until all differences between pairs of genotype groups are significantly different.

If all the genotypes have significantly different means in the first step the procedure ends at this step. Otherwise, the number of genotype groups in the final partitioning is less than the number of genotypes present at the start of the analysis. To measure the importance of the final model R^2 ^is determined, which is the proportion of the trait variation explained by the genotype groups. The significance of the model is estimated by permutation testing, generating a null distribution of R^2^. Bonferroni correction is applied for the number of factor combinations that have been tested. Factor combinations are selected if the explained variance R^2 ^by the combination is found to be significant. Analysis with RPM has been performed for irinotecan metabolism [32].

### Multifactor dimensionality reduction method

The multifactor dimensionality reduction method analyzes genetic and/or environmental effects on a dichotomic outcome variable (e.g. disease status) rather than a quantitative trait. MDR has been inspired by the CPM, but the approach differs in many perspectives.

From the total group of factors studied, MDR evaluates all possible N-factor combinations of genetic and/or discrete environmental factors. Each cell of the N-factor combination is assigned to either a low risk or high risk group. A certain threshold, defined as the ratio of cases to controls, determines the risk group to which a factor combination is assigned. For example, for all nine possible genotype combinations of each two loci combination the risk status is determined. If the threshold is set to one and the cell for a genotype combination contains more cases than controls, that genotype combination is determined as high risk. Thus, MDR assigns each combination (e.g. multi-locus genotype) within a N-factor combination to a high risk or low risk group, thereby constructing a new factor consisting of the two risk groups. The process of constructing a new factor as a function of two or more other factors is referred to as constructive induction and MDR can therefore be viewed as a constructive induction approach [38]. MDR evaluates the ability of this new factor to classify and predict disease status by multi-fold cross-validation.

Multi-fold cross-validation divides the observed data in equal subsets. One subset remains aside, the other subsets are used to build the model. The N-factor model with the lowest classification error is selected and for this model the remaining subset is used to obtain the prediction accuracy. By turns each subset is used to obtain the prediction accuracy for the best classifying model that has been build by the other subsets. The model with the highest prediction accuracy is selected as the best N-factor model.

Different numbers N of factors are evaluated. For each number of factors, multi-fold cross-validation is used to select the best classifying N-factor combination by measuring the prediction accuracy of the model. Cross-validation consistency (also discussed in the genetic programming optimized neural networks section) is another measure for selecting the best classifying N-factor combination: it is the number of times a N-factor combination is selected as the best model across all validation sets, divided by the number of validation sets. The N-factor model with the highest prediction accuracy and/or the highest cross-validity consistency is selected. If one best model is found with the highest prediction accuracy and another model with the highest cross-validation consistency, the most parsimonious model is chosen for describing the observed data. For example, if the best 2-factor combination model has the highest prediction accuracy and the best 3-factor combination model has the highest cross-validation consistency, the 2-factor combination model is selected. A permutation test is performed to obtain the statistical significance of the most predictive N-factor model. For each permuted dataset the best model is selected and the prediction accuracy or cross-validity consistency is determined. The p-value is obtained using the distribution of the prediction accuracy or cross-validity consistency under the null-hypothesis.

The MDR approach has been applied for example to case-control data of prostate cancer [39], type 2 diabetes [40], myocardial infarction [35], hypertension [41] and sporadic breast cancer [42].

### Features of the combinatorial methods

The combinatorial methods discussed above select from all factor combinations those factor combinations that best explain the outcome variable thereby solving the dimensionality problem. Because both CPM and MDR are computationally intensive procedures the number of factors to be analyzed by these methods is moderate. Selection methods to preselect factors can be used as a first step [38] and such filter methods are part of the MDR software [43]. These methods can be applied before using MDR, enabling the user of the MDR software to analyze large numbers of factors. Although RPM has the advantage that it relieves the computational intensity of CPM and thereby has the potential to analyze many interacting loci, the multiple testing problem is still a challenge for this method.

One of the merits of the combinatorial methods is their high power to identify high-order interactions between loci while main effects are not present [32,44]. The power of the MDR approach to detect gene-gene interactions in the absence of main effects was examined by Ritchie et al. [44]. Using simulated datasets, they studied 6 different models of interaction between two loci, including in the datasets noise due to 5 percent genotyping error, 5 percent missing data, 50 percent phenocopy and 50 percent genetic heterogeneity. Without noise factors, the power of the MDR method to detect the two-locus interaction for the 6 models was in between 80 and 100 percent. The drop in power due to genotyping errors, missing data or the combination of these noise factors was very small. Phenocopies had a large effect on the power for 4 models and genetic heterogeneity had the largest impact on the power for 5 of the 6 models. The power is reduced by phenocopies or genetic heterogeneity, because different combinations of factors causing the disease will decrease the prediction accuracy and cross validity consistency of a model [35]. The power to detect the interaction for each of the models was decreased to around 1 percent for the combination of phenocopies and genetic heterogeneity. If phenocopies are present, the power of the MDR approach can be increased if environmental factors causing the disease are included in the analysis. Environmental differences in the population can be assessed to define subgroups after which MDR can be applied to each group, or the environmental factors can be included in the MDR analysis. To account for genetic heterogeneity, cluster analysis of genetic markers can be employed (see background section). MDR analysis for the different clusters can be performed or the cluster status can be included as a covariate [44]. If the presence of genetic heterogeneity is not known beforehand the power for CPM, RPM and MDR is largely reduced.

As CPM, RPM and MDR select the model that has the best prediction of disease status, the model that contains the most information will be selected. Risk predictors contain more information than predictors correlated with the risk predictors and the power of these methods to detect risk SNPs will not be reduced when correlation between predictors is present. Software for CPM is not available, but a program for this method can easily be made by a competent statistical geneticist. Software implementing RPM is available from Culverhouse et al. [32]. Also, open-source software for RPM is currently under development [43]. MDR software, originally discussed by Hahn et al. [45], is freely available and is open-source [43]. A MDR Permutation Testing module to perform permutation testing is also freely available [43].

### Recursive partitioning methods

Recursive partitioning methods partition the total dataset recursively into smaller and more homogeneous subsets to fit models for predicting the value of a continuous or categorical outcome from many predictor variables. These models are called tree-based models as the splits of the data into more and more homogeneous subsets can be pictured by a tree graph [15]. Regression and classification trees are respectively applied to continuous and categorical outcome variables. Here, the application of random forests of classification trees to case-control data is discussed.

A tree is made up of internal and terminal nodes, with the first internal node called the root node that contains the total sample. The root node is split into two nodes to improve the homogeneity of the case group and control group compared to the root node. This split is based on a cut-off point of the predictor variable that partitions the total sample best into the two groups of cases and controls, for example a split based on a certain SNP with one subset containing wild-type homozygous and heterozygous individuals (genotypes AA and Aa) and the other subset containing homozygous mutant individuals (genotype aa). Each of these two nodes is split again, whereby splits are based on the predictor variable that improves the homogeneity of the resulting subsets (this predictor may differ for each node). A node that is not further split into two nodes is called a terminal node. A recursive partitioning method that can be used for selection of important predictors contributing to disease is the random forests approach.

### The random forests approach

In the random forests approach a group of tree-based models is used to select predictors with an important contribution to an outcome variable [16,46]. For each model, every split is based on a random selected subset of all predictors studied. More important predictors will discriminate best between cases and controls and will therefore be closer to the root node and present in most of the trees. On the other hand, less important predictors will be less present in the different trees and closer to the terminal nodes [46]. The random forests approach has been described in more detail by Lunetta et al. [16] and Bureau et al. [46].

### The prediction accuracy of the forest

For each tree in the forest, the total sample started with at the root node is generated by bootstrap sampling. With bootstrap sampling individuals are sampled from the observed population sample. The number of individuals in the bootstrap sample equals the number of individuals in the observed sample and because sampling is performed with replacement, some individuals can be present more than once in the bootstrap sample while other individuals are left out. The bootstrap sample is used to build the tree and the left-out individuals to obtain the prediction of the forest. The predictor values of a left-out individual determine which terminal node, or class, this individual is assigned to for a certain tree. The class to which most of the individuals of the bootstrap sample are assigned to is the predicted class of the tree for the left-out individual. The prediction for the forest is obtained by counting the predictions over the trees for which the individual was left out the bootstrap sample. The class with the most predictions is the prediction of the forest. In case-control data the prediction accuracy of the forest is given by the difference between the proportion of correct and incorrect classification of the left-out individuals. The prediction accuracy of the forest is used to obtain a measurement of importance of each predictor.

### The importance of predictors

Predictors that best classify the population into cases and controls are assumed to be important predictors of disease-status. The importance of a predictor is given by an importance index I_M _which denotes the importance of a predictor taken other predictors into account. The values of the predictor for which the index is computed need to be randomized for the left-out individuals to remove any association between the predictor and disease status. The importance index for predictor A is then the difference in prediction accuracy of disease status by the predictor vector and the same predictor vector with predictor A randomly permuted for the left-out individuals. Larger differences in prediction accuracy between the two predictor vectors indicate more important predictors. The random forests approach orders the predictors according to their importance. Computing the importance index can be extended to pairs of predictors whereby the predictor values of both predictors are permuted. Application of random forests to case-control data has been reported for asthma [46].

### Features of the random forests approach

As the random forests approach selects the most important predictors among all predictors, the dimensionality problem is circumvented, but the approach does not provide a cut-off value of the importance index to determine which predictors should be retained for further analysis [16]. The advantage of the random forests approach is that it is able to test many predictors. Permuting the predictor values for the left-out individuals does not only remove the association between the permuted predictor and the outcome variable, but also the interaction effects of the permuted predictor with other predictors, if present. Thereby, the interactions of the predictor with other predictors are captured in the importance index. Lunetta et al. [16] tested the performance of the random forests approach compared to Fisher's Exact test in ranking risk SNPs using simulated data. Genetic heterogeneity was included in the disease models. If interaction between two risk markers is present, the random forests approach has a better performance to rank these risk markers than univariate ranking methods because the importance of each marker involved in the interaction will increase. More interactions and larger groups that interact increase the relative performance [16]. Therefore, markers with weak main effect but significant interaction with other markers can be detected by the random forests approach. The joint importance of subsets of predictors can be tested for all markers if the size of the subset is small, but testing the joint importance for larger subsets to capture higher-order interactions becomes computationally unfeasible. As Province et al. [15] point out, recursive partitioning methods are able to detect genetic heterogeneity. This assertion is confirmed by the study of Lunetta et al. [16]. Genetic heterogeneity is handled because different models are fitted to subsets of the data defined by early splits in the trees [15,16]. Limited simulations suggest that correlated predictors are a problem for the random forests approach as it leads to a decrease of the predictor importance for each correlated risk SNP [16]. Software for the random forests is freely available and is open-source [47].

## Conclusion

An overview of the strengths and weaknesses of the methods discussed is given in table 1.

**Table 1:** Comparison of the different methods.

	Logistic regression	Neural networks		Set association	CPM	RPM	MDR	Random forests
							
		PDM	GPNN					
Outcome variable	dichotomous	categorical continuous	categorical continuous	dichotomous	continuous	continuous	dichotomous	categorical
Dimensionality	no	no	yes	yes	yes	yes	yes	yes
Number of predictors	few	moderate	many	many	moderate	many*	moderate†	many
Power to detect important effects	low	no info	high	high	high	high	high	high
Detection of interactions	no	yes	yes	no	yes	yes	yes	yes‡
Correlated predictors	no	no	yes	n.i.**	yes	yes	yes	no
Genetic heterogeneity	no	yes	yes	no	no	no	no	yes
Software available Open source	yes	yes no	no	yes yes	no	at request and under development	yes yes	yes yes

For the problems of dimensionality, correlated predictors and genetic heterogeneity yes and no indicate respectively that a method is able or not able to handle the problem. For detection of interactions when main effects are absent yes and no indicate respectively that a method is able or not able to detect interactions while main effects of the loci involved in the interaction are small or absent.

* RPM is subject to the multiple testing problem.

† MDR can analyze a moderate number of factors, but filter methods that are part of the MDR software can be applied before using MDR, enabling the user of the MDR software to analyze large numbers of factors.

‡ Interactions contribute to the importance of predictors.

** n.i.: not implemented, adjustment of the test statistics for correlation between markers is not implemented in the software.

The dimensionality problem is not solved by the method of logistic regression. Applying a parameter decreasing method within neural networks to select important predictors is a useful approach if moderate numbers of SNPs are tested. However, neural networks can not handle the dimensionality problem either if the number of predictors tested becomes too large. Logistic regression and neural networks are therefore less useful to approach association studies with large numbers of predictor variables. These methods can be applied to model the effects of a group of selected predictors, including interaction terms and other potential risk factors. For example in the two step approach coupled logistic regression can be used after the markers have been selected in the first step. Genetic programming optimized neural network is able to select and model important predictors from a set of predictors, but the performance of GPNN to detect important SNPs in the presence of large numbers of unrelated SNPs needs to be investigated.

Both the set association approach and random forests approach can handle a large number of predictors and are useful in reducing the large amount of predictors to those predictors with an important contribution to disease. Another argument for employment of the random forests approach is the possibility to detect the presence of genetic heterogeneity. The combinatorial methods are useful to give more insight in interaction patterns for sets of genetic and/or environmental predictor variables. CPM and RPM can be applied in the study of quantitative phenotypes underlying the disease of interest, MDR is useful for analyzing effects on disease status.

As each of the non-parametric methods has its strengths and weaknesses, genetic association studies should be approached by several methods. For genetic association studies using the case-control design to analyze complex diseases, the application of the set association approach in combination with the MDR and the random forests approach will most likely be a useful strategy to find the important genes and interaction patterns involved, as each of these methods approach the analysis of multiple SNP data differently. Similarities and differences in the results generated by these methods will provide valuable information whether selected SNPs are likely to contribute to disease by their main effects or whether gene-gene interactions play a role. Thus the combination of these methods will give more insight in the etiology of complex diseases. These methods can also be used in a multistep approach, discussed by Moore et al. [38], to detect and interpret interactions. In the first step of this approach a subset of important SNPs is selected from the total number of SNPs. The set association approach and/or random forests could be applied as method for selection of important predictors. The next step is to apply a constructive induction approach to construct from this subset of SNPs a new factor consisting of high risk and low risk genotype combinations. MDR can be used at this second step as a constructive induction approach. The ability of this constructed factor to classify and predict disease status is evaluated in the third step, for example by multi-fold cross-validation which is also implemented in the MDR approach. Besides detecting statistical interactions this multistep approach provides the means to statistically interpret the detected interactions in the fourth step. At this last step visual tools can be used for model interpretion. This multistep approach is flexible as at each step many different methods can be used [38].

More statistical methods to analyze multiple SNPs in relation to complex diseases are becoming available. What the features of other newly developed methods for analysis of multiple SNPs will be has to be studied and compared to the methods discussed in this commentary. Also, applications of the methods in genetic association studies will have to be performed in order to examine their practical value for the field of genetic epidemiology.

In this commentary the strengths and weaknesses of methods to approach the statistical challenge to detect gene-gene interactions associated with the disease or disease related outcome of interest have been discussed. However, these methods test interactions statistically, which is only a first step in the unravelling of the interacting underlying biological mechanisms. The biological interpretation of statistically detected gene-gene interactions is not straightforward and forms another challenge for genetic epidemiologists. Statistical interaction is detected on the population level by relating genotype information to interindividual differences in phenotype while biological interaction is the result of physical interaction of biomolecules which takes place at the individual level [2]. To address this challenge, Moore et al. propose the application of systems biology (a synthesis of multiple disciplines) to unicellular organisms, reasoning that understanding of the relationship between statistical and biological interaction in these organisms will reveal some basic underlying principles and thereby will help to understand how statistical interaction is related to human complex diseases [2].

In conclusion, statistical methods have been developed that enable genetic epidemiologists to detect important genetic and/or environmental predictors associated with disease or disease related variables. These methods have different strengths and weaknesses. Applying a combination of these methods will provide insight in the main effects and interaction patterns involved in the etiology of complex diseases.

## Authors' contributions

AGH drafted the manuscript and prepared the final version of the manuscript. JMAB, NN, ECMM, DLA and EJMF participated in writing the manuscript. All authors have read and approved the final manuscript.
